# Determining Carina and Clavicular Distance-Dependent Positioning of Endotracheal Tube in Critically Ill Patients: An Artificial Intelligence-Based Approach

**DOI:** 10.3390/biology11040490

**Published:** 2022-03-23

**Authors:** Lung-Wen Tsai, Kuo-Ching Yuan, Sen-Kuang Hou, Wei-Lin Wu, Chen-Hao Hsu, Tyng-Luh Liu, Kuang-Min Lee, Chiao-Hsuan Li, Hann-Chyun Chen, Ethan Tu, Rajni Dubey, Chun-Fu Yeh, Ray-Jade Chen

**Affiliations:** 1 Department of Medicine Research, Taipei Medical University Hospital, Taipei 11031, Taiwan; lungwen@tmu.edu.tw; 2 Department of Information Technology Office, Taipei Medical University Hospital, Taipei 11031, Taiwan; 3 Graduate Institute of Data Science, College of Management, Taipei Medical University, Taipei 11031, Taiwan; 4 Professional Master Program in Artificial Intelligence in Medicine, College of Medicine, Taipei Medical University, Taipei 11031, Taiwan; traumayuan@gmail.com; 5 Department of Surgery, Da Chien General Hospital, Miaoli 36052, Taiwan; 6 Department of Emergency Medicine, Taipei Medical University Hospital, Taipei 11031, Taiwan; 992001@h.tmu.edu.tw; 7 Department of Emergency Medicine, School of Medicine, College of Medicine, Taipei Medical University, Taipei 11031, Taiwan; 8 Taiwan AI Labs, Taipei 10351, Taiwan; wlogsky666@gmail.com (W.-L.W.); henryhsu9238@berkeley.edu (C.-H.H.); liutyng@iis.sinica.edu.tw (T.-L.L.); tony.lee@ailabs.tw (K.-M.L.); chaioshuan@ailabs.tw (C.-H.L.); hannchyun.chen@ailabs.tw (H.-C.C.); ptt@ailabs.tw (E.T.); 9 Division of Cardiology, Department of Internal Medicine, Taipei Medical University Hospital, Taipei 11031, Taiwan; 10 Division of Infection Diseases, Department of Internal Medicine, Taipei Medical University Hospital, Taipei 11031, Taiwan

**Keywords:** endotracheal intubation, endotracheal tube, chest X-ray, carina, clavicle

## Abstract

**Simple Summary:**

Endotracheal intubation (ETI) is employed for maintaining the airway patency of in critically ill patients during mechanical ventilation. Generally, ETI is conducted under anesthesia in the intensive care unit, during which the endotracheal tube (ETT) is inserted at a particular depth into the trachea, and a malpositioned ETT may result in hazardous consequences, such as a collapsed or hyperinflated lung. Therefore, we proposed a deep learning-based CNN approach, combined with four key point annotations on chest radiographs (tracheal tube end, carina, and left/right clavicular heads), which demonstrated significant sensitivity, specificity, and accuracy for recognizing and localizing the ETT tip on chest radiographs. These findings may assist in radiographic confirmation of precise ETT placement and help in ruling out other etiologies of respiratory failure.

**Abstract:**

Early and accurate prediction of endotracheal tube (ETT) location is pivotal for critically ill patients. Automatic and timely detection of faulty ETT locations from chest X-ray images may avert patients’ morbidity and mortality. Therefore, we designed convolutional neural network (CNN)-based algorithms to evaluate ETT position appropriateness relative to four detected key points, including tracheal tube end, carina, and left/right clavicular heads on chest radiographs. We estimated distances from the tube end to tracheal carina and the midpoint of clavicular heads. A DenseNet121 encoder transformed images into embedding features, and a CNN-based decoder generated the probability distributions. Based on four sets of tube-to-carina distance-dependent parameters (i.e., (i) 30–70 mm, (ii) 30–60 mm, (iii) 20–60 mm, and (iv) 20–55 mm), corresponding models were generated, and their accuracy was evaluated through the predicted L1 distance to ground-truth coordinates. Based on tube-to-carina and tube-to-clavicle distances, the highest sensitivity, and specificity of 92.85% and 84.62% respectively, were revealed for 20–55 mm. This implies that tube-to-carina distance between 20 and 55 mm is optimal for an AI-based key point appropriateness detection system and is empirically comparable to physicians’ consensus.

## 1. Introduction

Endotracheal tube (ETT) positioning in critically ill patients is highly crucial while managing airway protection or mechanical ventilation. For such patients, endotracheal intubation (ETI) is generally conducted under anesthesia in the intensive care unit (ICU), and a malpositioned ETT may result in hazardous outcomes, such as a collapsed or hyperinflated lung [1]. Reportedly, the incidence of ETT malposition and associated complications ranges from 0.5 to 7%, respectively [2]. Apart from absolute indicators such as upper airway obstruction, the decision and timing of intubation for specific patients may vary. Hence, a clinician needs to balance the emergency intubation risks against delaying intubation in a patient to mitigate and modify the airways management plan at the bedside.

Radiographic evaluation of ETT positioning is most commonly carried out, particularly among ICU patients, on a reliable chest X-ray (CXR) which is inexpensive and can be promptly obtained at any location of the hospital [1]. When correctly placed, the tip of the ETT should be positioned in the mid-tracheal region, or halfway between the clavicles and the carina. Upper or lower chin position impacts the ETT depth, which renders it to be higher or deeper, respectively [3]. So, the ETT positioning must be precise to suppress the incidence of complications including tracheal damage and hyperinflation of the lung [1]. Hence, using chest X-ray images (CXR) and artificial intelligence (AI), this study aimed at predicting the appropriateness of the ETT position by evaluating the relative distribution of the four key points (i.e., tracheal tube end, carina, and left/right clavicular heads). 

In recent years, AI technology has facilitated medical image analysis and biomedical signal processing. Its use in object detection and recognition in X-rays is an emerging area. So far, there is limited applicability of AI models in the clinical practice of ICU. In the ICU, AI may assist clinicians on diagnostic, prognostic, and curative levels to revamp patient outcomes. Currently, it has been used in detecting therapeutic tubes and catheters [4,5], Deep learning, a form of AI, is being increasingly employed in the processing of medical images of the eyes, brain, breast, chest, musculoskeletal system, pelvis, and abdomen [6,7]. Deep learning could automate the detection of thoracic disorder in CXR [8]. Of these, an approach known as the convolutional neural network (CNN) was adopted in this work as the core computational model to extract image data features at different levels by utilizing multiple processing layers. 

The underlying model architecture mainly comprised two parts. The encoder was implemented based on a pre-trained DenseNet121 to transform images into embedding features, while the decoder was a CNN subnetwork boosted with attention mechanisms to generate the probability distributions of the four key points. Through the proposed CNN model, we attempted to characterize the ETT position with the reference to sensitivity, specificity, and accuracy.

## 2. Materials and Methods 

### 2.1. Datasets

This research employed the datasets from Taipei Medical University Hospital (TMUH), one of the leading teaching hospitals in Taiwan. Following the approval of the institutional review boards of TMUH (IRB number: N202007011), 427 images from 183 patients were obtained from the TMUH database and were split into the training and validation sets, so that CXR images from the same patient would only appear in either the training or validation set. Specifically, we randomly (patient-wise) split the data (80:20) into training (*n* = 348) and validation sets (*n* = 79).

**Figure 1:**
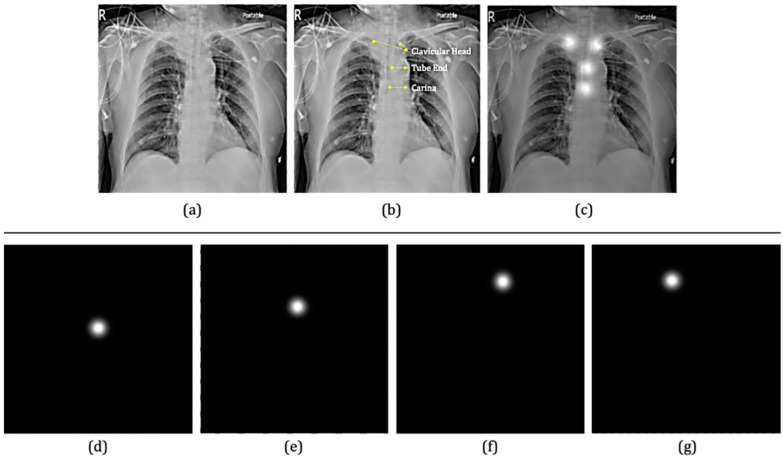
An example of the image and key points annotations. (**a**) The chest radiograph. (**b**) The coordinates (x, y) of left/right clavicular heads, tube end, and carina were marked by one physician. (**c**) The two-dimensional (2D) Gaussian function of these four key points is superimposed over the chest radiograph. (**d**–**g**) 2D Gaussian function of each key point, which serves as the ground truth for the model.

Additionally, 42 chest X-ray images from 34 patients were collected as a test dataset to evaluate the performance of the developed model. All of the digital radiographic images were in DICOM format with free-text radiology reports. These datasets were further annotated by a group of certified physicians. According to the clinical practice of ETT positioning evaluation, four key points, including tracheal tube end, carina, and left/right clavicular heads (Figure 1) were marked for each chest X-ray image in the training, validation, and test sets. Each point was represented by (x, y) coordinates. 

In agreement with the previous report [9], the left/right clavicular heads were included as these locations with the reference to trachea might help to determine whether the patient’s head was in a neutral position during the chest radiography. Since the ETT position varies with the neck position and rotation, we also included the mandible and C7 vertebra as important indicators [10]. For the training and validation sets, each chest X-ray was annotated by one physician, whereas for the test set the three physician-based consensuses about four key points were obtained. The consensus of each key point was defined as the midpoint of the three marked key points. Besides those four key points, the consensus on the appropriateness of the ETT position was also derived for the test set. Label 1 represents an adequate ETT position (normal) while 0 indicates an abnormal position. In total, the ratio of the numbers of normal images and abnormal ones in the test dataset was 2:1, and 40 out of 42 images were categorized as mandible above the C7 group, indicating that the mandible of the patient was above C7 in the radiograph (Table 1).

Besides the standard machine learning process which used training, validation, and test sets to learn and evaluate the model, an isolated clinical evaluation set was collected to demonstrate the level of agreement between the model and a physician in the common clinical practice. In this set, there were a total of 103 images from 35 patients. To simulate the condition of applying the model for the appropriateness of ETT position in clinics, the images were randomly partitioned into five portions, each of which was reviewed solely by one of a group of three certified physicians. Each image was annotated as normal (appropriate ETT position) or abnormal. No consensus was made, and no key point was marked in this set. All of these images were categorized as mandible above the C7 group and the ratio of normal images (*n* = 79) to abnormal ones (*n* = 24) was 3:1. 

### 2.2. Data Preprocessing and Augmentation

All of the images were normalized to a 0–1 abundance scale and then resized to 512 × 512 for modeling. Each key point (x, y) was transformed to a two-dimensional Gaussian of constant variance (σ=10 pixels), centering at coordinates marked by the physicians. During the model training, data augmentation was performed via random scaling and random rotation to prevent the model from overfitting the training set. For random scaling, the range was randomly selected between 90% (zoom-in) and 125% (zoom-out), whereas the range of rotation was set between −45 degrees and 45 degrees.

### 2.3. Modeling Framework

**Figure 2:**
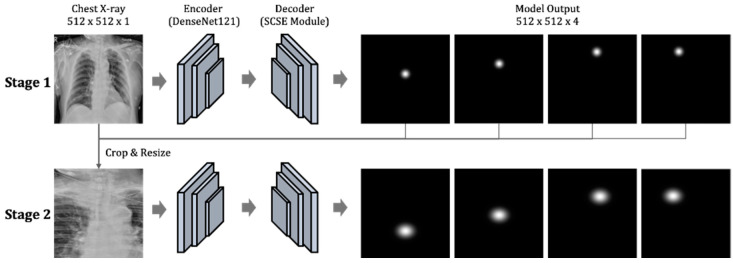
The two-stage key point detection model.

To automate the evaluation of the ETT position with chest X-rays, our proposed framework consists of two main components. The first component represents a two-stage key point detection model, which detects the four key points, namely tube end, tracheal carina, and left/right clavicular heads, and subsequently fine-tunes the locations of the tube end and tracheal carina (Figure 2). 

The inputs and labels in the first component were two-dimensional data with the size of 512 × 512. These inputs were preprocessed chest X-ray (CXR) images, and the labels were two-dimensional Gaussian distributions. The second component predicts the appropriateness of the ETT position based on the four detected key points from the first component. Then, a set of clinical parameters would be applied to these estimated coordinates to derive the appropriateness of the ETT position. The details of the first and second components of our model are described in the next sections.

#### 2.3.1. The First Component: Two-Stage Key Point Detection Model

As illustrated in Figure 2, we used DenseNet121 (pre-trained on ImageNet) as the encoder to transform images into embedding features. The decoder to generate probability distributions of the four key points was composed of three convolutional layers with spatial and channel squeeze and excitation (SCSE) module [11] followed by a 1 × 1 convolutional layer. Networks in our encoder-decoder structure were identical in both stages. Input (512 × 512 × 1) and output (512 × 512 × 4) formats for each stage were the same. The value of four in the third (channel) dimension of the output corresponded to the number of key points to be detected. Each feature map in that dimension represented the estimated 2D probability distribution of the key point coordinates (x, y), which were derived by accessing the location of the highest probability.

The input for the second stage was obtained by cropping and resizing each source image with respect to the four key points. During training, the key points used to crop the source images were the ones marked by the physicians. For the inference, we used the predicted key points from the first stage to obtain the cropped images. We employed the PyTorch framework (version: 1.8.1) throughout the training process. Each stage was trained separately with Adam optimizer, with a learning rate of 0.0001 and a batch size of 8 for 1000 epochs on a GeForce GTX 2080 Ti graphics processing unit (GPU). We used binary cross-entropy as the loss function for appropriateness classification and L1 distance as the loss between the predicted key point and the ground truth heatmaps.

#### 2.3.2. The Second Component: Appropriateness Prediction

**Figure 3:**
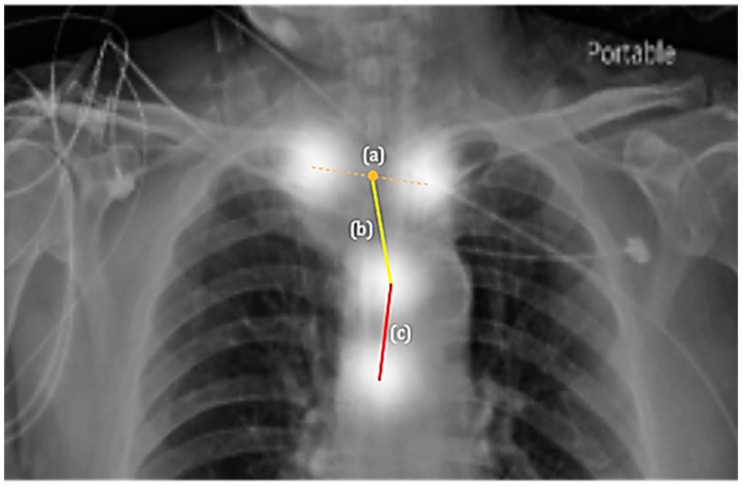
The estimated distances from key point predictions. (**a**) The midpoint of clavicular heads. (**b**) Tube-to-clavicle distance. (**c**) Tube-to-carina distance.

With the four key points, we further considered the clinical parameters for appropriateness prediction and the estimated distances from the tube end to the tracheal carina and the midpoint of clavicular heads (Figure 3). These parameters included the following: tube-to-carina distance between (i) 30 mm to 70 mm, (ii) 30 mm to 60 mm, (iii) 20 mm to 60 mm, or (iv) 20 mm to 55 mm, where an estimated distance within each specific range would be considered normal (Table 2). Using the training and validation data, we learned to detect the four key points and hypothesized the four distance ranges based on related work and experts’ domain knowledge. On the other hand, the consensus by the three physicians also implicitly defined an underlying range in deciding the position appropriateness. Thus, by assuming in turn each of the four distances as the decision rule, the one that yields the best performance on the test set could be the closest to the range implied by the consensus process. We also experimented with the tube-to-clavicle distance along with these four sets of parameters (Table 3). Specifically, an ETT position would be considered normal when its tube-to-carina distance was within the parameters and its tube-to-clavicle distance was greater or equal to zero (i.e., the tube was below the midpoint of the clavicular heads). Additionally, for appropriateness prediction, we averaged the binary predictions (0 or 1), which were derived from the combinations generated by the following parameters: (i) the lower bound of tube-to-carina distance ranged from 20 mm to 30 mm with an interval of 1 mm, (ii) the upper bound of the tube-to-carina distance ranged from 55 mm to 70 mm with an interval of 1 mm, (iii) the tube-to-clavicle distance ranged from −5 mm to 5 mm with an interval of 1 mm. There was a total of 1936 combinations, each of which served as a set of parameters for appropriateness prediction, and the averaged predictions were compared to ground truths to evaluate the model performance on the test dataset. Unlike the use of expert knowledge that limits the range candidates of the tube-to-carina distance to four, the general setting here considers a total of 1936 combinations and yields a weighted appropriateness prediction. The weighted scheme was used for deriving the ROC curve and AUC.

### 2.4. Statistics

For the two-stage keypoint detection, we measured the mean absolute distance in mm between the predicted key points and the ground truths. The model efficacy was determined by performance indices in terms of sensitivity and specificity, which were compared with the consensus of the physicians. For appropriateness predictions, the receiver operating characteristics (ROC) curve and the area under the ROC curve (AUC) was measured and the optimal sensitivity and specificity were derived by using the Youden index. 

## 3. Results

### 3.1. Key Point Detection

**Figure 4:**
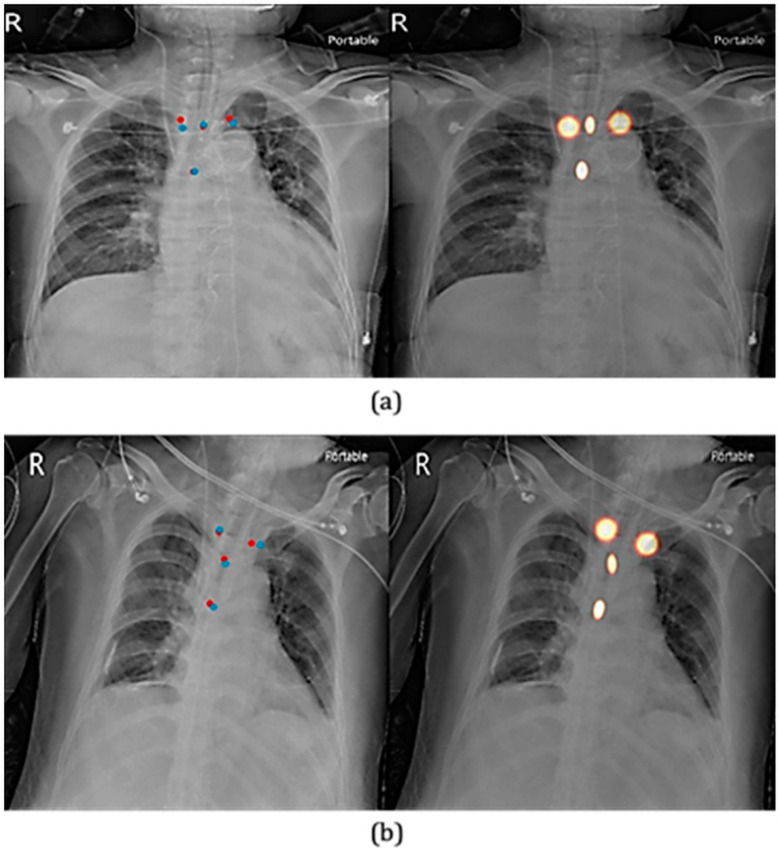
Visualizations of predicted key points. (**a**) Mandible above C7. (**b**) Mandible below C7. The right panel of each row illustrates the predicted heatmaps of key points, while the left panel shows the locations of predicted key points (in blue) and ground truths (in red).

Specifically, we found the mean absolute distance of tube end predictions and tracheal carina predictions as 3.04 mm and 2.42 mm, respectively. In comparison, the mean absolute distances of the left (3.77 mm) and right clavicular head predictions (3.59 mm) were slightly greater, indicating that these two points were more difficult to locate, most likely due to the uncertainty in the annotations. Examples of the visualizations of predicted key points superimposed over the original image are shown in Figure 4.

### 3.2. Appropriateness Prediction

To predict the appropriateness of the ETT position, we considered four sets of parameters to the tube-to-carina distances derived from the predicted tube end and carina. These results have been reported in Table 2. In the case of sensitivity, that derived from the distance between 20 mm/30 mm to 60 mm was higher (57.14%) than that between 30 mm to 70 mm (35.71%). The value reached the highest (71.42%) as the maximal distance to be considered for the adequate position was reduced to 55 mm. This indicates that the tube-to-carina distance between 20 mm to 55 mm is optimal for an AI-based key point appropriateness detection system and may be comparable to physicians’ consensus. Moreover, the improved performance with only a 5 mm difference on the maximal tube-to-carina distance could be attributed to the parameter making up the detection errors on the tube end (3.04 mm) and the carina (2.42 mm). This improvement and the ensuing sensitivity-specificity trade-off would be the gray area of subjective interpretation on the ETT position in the distance between 55 mm and 70 mm to the carina. 

Moreover, we further evaluated the performance by adding tube-to-clavicle distances, an additional parameter, which has been demonstrated in Table 3. 

Our result showed greatly improved sensitivities of all of the parameters, especially, the 30 mm to 70 mm. This appropriateness prediction model represents higher sensitivity without significantly compromising the specificity and corroborates our previous findings in Table 2, indicating the possible uncertainty in the ETT position as the tube is located near the clavicle heads. 

### 3.3. Receiver Operating Characteristic (ROC) of the Prediction Model

**Figure 5:**
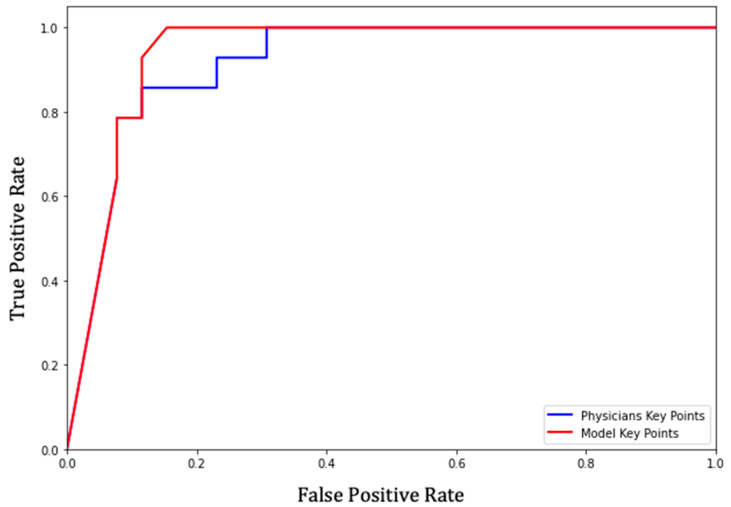
ROC curve for appropriateness prediction of the model. The red and blue colored lines indicate the key point curves of the model and physicians, respectively. ROC, receiver operating characteristic.

To predict the appropriateness of the ETT position, we applied each combination of parameters on the key points. Thereafter, we averaged out binary appropriateness predictions for deriving the ROC curve (Figure 5) and AUC. 

We also applied the same process on the key points of physicians’ consensus, to produce a comparative result. Our prediction model showed an area under curve (AUC) score of 0.9381, which is very close to physicians’ consensus of 0.9175. Further, using the Youden index, we found the optimal sensitivity and specificity of our model as 100.00% and 84.62%, respectively.

### 3.4. Appropriateness Prediction on the Clinical Evaluation Dataset

**Figure 6:**
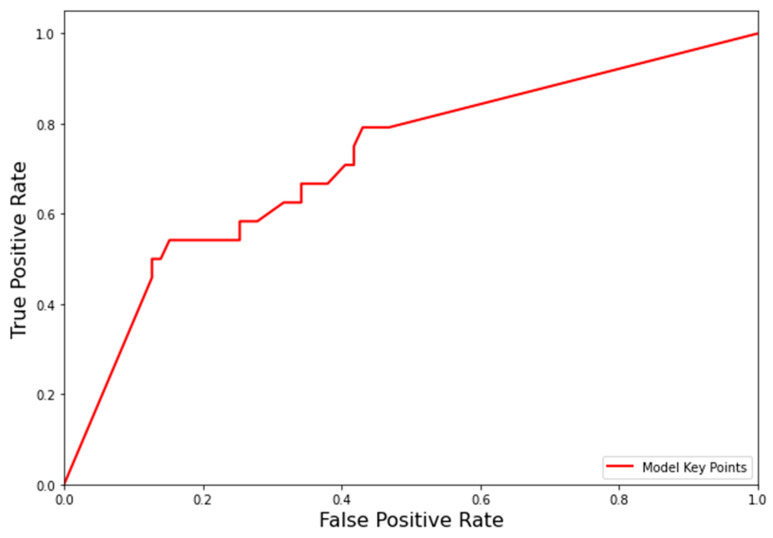
ROC curve for appropriateness prediction of the model based on the clinical dataset evaluation. The red-colored line indicates the key point curves of the model. ROC, receiver operating characteristic.

To demonstrate the level of agreement in the common clinical setting, we followed the same above-mentioned process to generate appropriateness prediction on the clinical evaluation set. The ROC curve has been represented in Figure 6, which showed that our model had an AUC score of 0.7181 on this isolated clinical evaluation set, and the optimal sensitivity and specificity with Youden Index were 79.17% and 56.96%, respectively. This indicates that the model did not have a high level of agreement with a single physician, and inconsistency in the subjective appropriateness annotations may be an issue.

## 4. Discussion

This is the first report on carina and clavicular distance-dependent identification and localization of positioning of ETT in critically ill patients. In this study, based on the clinical practice of ETT positioning evaluation, four key points including tracheal tube end, carina, and left/right clavicular heads, were identified for each chest X-ray image. With respect to trachea, the left and right clavicular heads were included as these might assist in localizing the ETT positioning. Since the ETT position varies with neck position and rotation, we also included mandible and C7 as important indicators [10]. To date, a few studies have investigated various parameters for determining ETT insertion and final positioning. Some of them are based on vocal cord-carina distance and tracheal length [12], stature, and incisor manubrio-sternal joint length [13]. Further, an individual’s height [14] and various anatomical landmarks have also been employed to predict airway length resulting in varying outcomes [13]. A seminal study reported that the tracheal midpoint corresponds internally to a line drawn between the medial heads of the clavicles and hence clavicles have also been employed as a reference point [15]. When placed correctly, the tip of the ETT must be positioned in the mid-tracheal region, halfway between the inferior clavicle and carina, which also coincides with our clinical rule [16]. However, Blayney et al. found the inconsistent position of the clavicle on chest X-ray and suggested using the first thoracic vertebra as a marker for correct tip placement [17]. Taken together, irrespective of the reference point used, the tube must always be positioned at a safe distance from the carina to avoid accidental endobronchial intubation. 

According to Goodman’s criteria, the ETT should be ideally placed in the mid-trachea approximately 50 mm above carina with the patient’s head in a neutral position, considering neck extension or flexion values of about 20 mm while moving downwards or upwards [18,19]. It also recommends that the mean tracheal tip to a carinal distance of 40 mm (range: 30–50 mm) may avert carinal impingement and endobronchial intubation [20]. In a study, the fiberoptically measured optimal placement of tube 2.5–4 cm above carina has been documented [21]. In concord with this report, a seminal randomized trial on 160 patients, recommended the 20/22 cm rule (i.e., inserting tubes to 20–21 cm in women and 22–23 cm in men, with the distal ETT tip less than 2.5 cm away from the carina to avert inadvertent endobronchial intubation) [22]. In contrast to adults, the ETT tip to the tracheal carina has been reported as 1.57 cm in the younger individuals [23]. Notably, the distance from the ETT tip to just above tracheal carina in the infants has been suggested in the range of 0.2 and 2 cm, depending on their age [24,25]. However, in a randomized controlled trial on newborn infants, the correct tube position just above carina has been employed as less than 0.2 cm [26]. Considering this evidence, out of the four distance parameters of tube-to-carina, our prediction model showed the highest sensitivity of 71.42% for 20 mm to 55 mm. Even based on tube-to-carina and tube-to-clavicle distance, the highest sensitivity, and specificity of 92.85% and 84.62%, respectively, was revealed for 20 mm to 55 mm. This result is in line with Goodman’s criteria, and implies that a tube-to-carina distance between 20 mm to 55 mm is optimal, in the safe limit, and comparable to physicians’ consensus. The less satisfactory results on the other three distance ranges do not imply the ineffectiveness of our model, but their deviation from the underlying range criterion embodied in the three physicians’ consensus. With the 20–55 mm range for classifying the tube-to-carina distances, our model can effectively determine whether the position of the tube end is proper or not. A recent study using the MIMIC Chest X-ray database also employed CNN-based algorithms to identify and localize the ETT position relative to the carina on chest radiographs [27]. Their distal ETT tip was approximated within a median error of 4.6 mm and 6.0 mm from ground-truth annotations respectively, which is comparatively higher than our results. However, this study did not include clavicular distance as a parameter and their ETT demonstrated sensitivity, specificity, accuracy, and AUC of 0.9737, 0.9689, 0.9714, and 0.9958, respectively. Our prediction model showed AUC score of 0.9381, which is almost coincides with physicians’ consensus of 0.9175. Besides, a computer-aided detection technique has also been used to estimate the ETT positioning with a sensitivity and accuracy of 85% and 81% for ETT detection and localization within 10 mm of ground-truth annotations of testing images [28]. Using a fully convolutional CNN model with combined real and synthetic data, the entire course of the ETT has been localized. However, it lacks the location of the distal tip of ETT relative to carina [29]. Apart from detecting the ETT tube on chest radiographs, previous studies have used deep CNN to the Indiana, JSRT, and Shenzhen datasets to localize various ranges of abnormalities, in particular, cardiomegaly with the highest accuracy of 92% and highest AUC of 0.9408 for detecting cardiomegaly [30]. Further, a 121-layer CNN-based on the NIH dataset has also been used for predicting pneumonia from frontal-view chest X-ray images [31]. Notably, the recent years have evidenced an increasing number of studies on ICU-AI models, mostly focusing on predicting complications, mortality, and improving prognostic models [32]. Using large population datasets, AI has mainly been employed in critical care to predict length of stay, ICU readmission and mortality rates, complications and risk stratifications [33]. Lately, a systematic review revealed that ML models could accurately predict onset of septicemia in ICU patients [34]. In a very important study, deep learning has been found as effective as senior radiologists in detecting lung nodules in CXRs and CT scans [35]. These studies provided the basis for conducting extensive deep learning-based prediction models to evaluate ETT position through utilizing chest radiographs of ICU patients. 

Besides various outcomes, this study possesses some limitations. Our results may be valid only for the Taiwanese population and may not apply to other ethnic populations. However, recent studies have also documented no clinically major difference between the tracheal diameter of the adult Chinese and Caucasian patients [36]. Moreover, the impact of ethnicity on tracheal diameter has been found small, when adjusting age, sex, height, and weight. So, we also assume that this model has the potential to generalize. It is of note that the normal/abnormal label was tagged by the physician, while the prediction model employed rules of the clinical standard. Therefore, the prediction of four key points may not be accurate due to the difference between the judgment of physicians and the clinical rules. Another limitation includes that clinician may judge the intubation check with patients’ body shape, while the computer only considers the distance between tube/clavicle and carina. The appropriateness of the ETT position was decided by physicians as per clinical experience. A consensus was taken for the first test set to determine the model performance. However, for the second test set, no model training was required. That is why only clinical validation was carried out with a standard single opinion, which was made by a trained physician, a representative of many physicians’ consensus. 

We would also like to highlight that our model has been evaluated based on test set and the clinical evaluation set. It could be noticed from our data that the ground truths of both sets are not of the same quality. The ground truth of each image in the test set was obtained by the consensus of three experienced physicians, while in the clinical evaluation set, it was decided by only a single physician. The design of these two experiments is two-fold. Firstly, we aimed to demonstrate that the proposed AI-based model can yield good performance with respect to more reliable ground-truth annotations (i.e., the consensus-based test set). Secondly, we attempted that without explicitly marking the four key points, the task to decide whether the position of an ETT end in CXR is appropriate or not is at times challenging even to an experienced physician. Thus, the less-satisfactory performance on the clinical evaluation set is largely due to the subjective variations on deciding each ground truth based on the decision of only one physician, rather than the inefficiency of the proposed model. Hence, in ICU, the detection of an inappropriate position of tracheal tube end is possible by the physician-in-charge. However, the proposed model should be useful in aiding such decisions with enhanced accuracy. In the emerging studies, the subjective opinions of clinical experts are the traditional basis of clinical practice [37] sometimes using consensus development [38]. In line with this, a previous study also demonstrated the majority vote of three cardiothoracic specialty radiologists as ground truth [39]. Further, our training dataset may include multiple CXR images of a patient, which were not taken consecutively. It is of note that a patient is likely to be subjected to CXR examinations several times during a single ICU stay, and the time intervals between these CXRs are usually at least 12 h. In addition, the position of tracheal tube end of a patient in CXR is not invariably fixed and could be changed due to different head positions or CXR viewing angles. These conditions highly reduce the impact of repeated CXRs images on model performance.

Our prediction suggests the need for extensive research and consensus on the ideal position of the ETT as even a minor length difference may have an adverse impact on respiratory morbidity, particularly in the neonates or infants [40]. Using currently manufactured tubes with whole centimeter markings, the adjustments of less than 1 cm remain challenging. Therefore, we suggest that manufacturing an ETT with ½ cm markings may aid in more accurate placement.

## 5. Conclusions

Our CNN approach combined with four key points annotations on chest radiographs (tracheal tube end, carina, and left/right clavicular heads) evidence a significant sensitivity, specificity, and accuracy for both identification and localization of the ETT tip on chest radiographs. Our results may support the radiographic confirmation of precise ETT placement and could help in ruling out other etiologies associated with respiratory failure. In the future, the clinical integration of deep learning tools, including user interface optimization to suppress workflow disruption and revamp overall clinical response time may be targeted. This system of machine learning and neural networks could handle enormous volumes of data in bringing positive changes in clinical decision-making processes, such as the automated interpretation of medical images. Additionally, it would reduce the medical staff’s workload and enhance patient safety.

## Figures and Tables

**Table 1:** The patient position-based test dataset.

	(*n*) Images in Test Dataset Normal/Abnormal
Mandible above C7	26/14
Mandible below C7	2/0

**Table 2:** Appropriateness prediction performance based on tube-to-carina distance on the test dataset. N/A, not applicable. Distances are represented in millimeters (mm).

	Test Dataset (Normal/Abnormal)			
	Mandible Above C7 (26/14)		Mandible Below C7 (2/0)	
**Parameters**	**Sensitivity (%)**	**Specificity (%)**	**Sensitivity (%)**	**Specificity (%)**
30 ≤ Distance < 70	35.71	100.00	N/A	100.00
30 ≤ Distance < 60	57.14	100.00		100.00
20 ≤ Distance < 60	57.14	100.00		100.00
20 ≤ Distance < 55	71.42	92.30		100.00

**Table 3:** Appropriateness prediction based on tube-to-carina and tube-to-clavicle distance (≥0 mm) using the test dataset. Distances are represented in millimeters (mm).

	Test Set (Normal/Abnormal)			
	Mandible Above C7 (26/14)		Mandible Below C7 (2/0)	
**Parameters**	**Sensitivity (%)**	**Specificity (%)**	**Sensitivity (%)**	**Specificity (%)**
30 ≤ Distance < 70	71.42	88.46	N/A	100.00
30 ≤ Distance < 60	85.71	88.46		100.00
20 ≤ Distance < 60	85.71	88.46		100.00
20 ≤ Distance < 55	92.85	84.62		100.00

## Data Availability

The data presented in this study are available on request from the corresponding author.
